# Links Between Metabolic and Structural Changes in the Brain of Cognitively Normal Older Adults: A 4-Year Longitudinal Follow-Up

**DOI:** 10.3389/fnagi.2019.00015

**Published:** 2019-01-15

**Authors:** Christian-Alexandre Castellano, Carol Hudon, Etienne Croteau, Mélanie Fortier, Valérie St-Pierre, Camille Vandenberghe, Scott Nugent, Sébastien Tremblay, Nancy Paquet, Martin Lepage, Tamàs Fülöp, Éric E. Turcotte, Isabelle J. Dionne, Olivier Potvin, Simon Duchesne, Stephen C. Cunnane

**Affiliations:** ^1^Research Center on Aging, Centre Intégré Universitaire de Santé et de Services Sociaux de l’Estrie (CIUSSS) de L’Estrie—Centre hospitalier Universitaire de Sherbrooke (CHUS), Sherbrooke, QC, Canada; ^2^Centre de Recherche sur le Vieillissement (CERVO) Brain Research Centre, Centre Intégré Universitaire de Santé et de Services Sociaux (CIUSSS) de la Capitale-Nationale, Québec, QC, Canada; ^3^School of Psychology, Université Laval, Québec, QC, Canada; ^4^Department of Pharmacology and Physiology, Université de Sherbrooke, Sherbrooke, QC, Canada; ^5^Sherbrooke Molecular Imaging Center, Université de Sherbrooke, Sherbrooke, QC, Canada; ^6^Department of Nuclear Medicine and Radiobiology, Université de Sherbrooke, Sherbrooke, QC, Canada; ^7^CR-Centre hospitalier Universitaire de Sherbrooke (CHUS), Centre Intégré Universitaire de Santé et de Services Sociaux de l’Estrie (CIUSSS) de l’Estrie—Centre hospitalier Universitaire de Sherbrooke (CHUS), Sherbrooke, QC, Canada; ^8^Department of Medicine, Université de Sherbrooke, Sherbrooke, QC, Canada; ^9^Faculty of Physical Activity Sciences, Université de Sherbrooke, Sherbrooke, QC, Canada; ^10^Department of Radiology, Université Laval, Québec, QC, Canada

**Keywords:** aging, brain energy metabolism, cognition, glucose, insulin resistance, ketones

## Abstract

We aimed to longitudinally assess the relationship between changing brain energy metabolism (glucose and acetoacetate) and cognition during healthy aging. Participants aged 71 ± 5 year underwent cognitive evaluation and quantitative positron emission tomography (PET) and magnetic resonance imaging (MRI) scans at baseline (*N* = 25) and two (*N* = 25) and four (*N* = 16) years later. During the follow-up, the rate constant for brain extraction of glucose (K_glc_) declined by 6%–12% mainly in the temporo-parietal lobes and cingulate gyri (*p* ≤ 0.05), whereas brain acetoacetate extraction (Kacac) and utilization remained unchanged in all brain regions (*p* ≥ 0.06). Over the 4 years, cognitive results remained within the normal age range but an age-related decline was observed in processing speed. K_glc_ in the caudate was directly related to performance on several cognitive tests (*r* = +0.41 to +0.43, *all* *p* ≤ 0.04). Peripheral insulin resistance assessed by the homeostasis model assessment of insulin resistance (HOMA-IR) was significantly inversely related to K_glc_ in the thalamus (*r* = −0.44, *p* = 0.04) and in the caudate (*r* = −0.43, *p* = 0.05), and also inversely related to executive function, attention and processing speed (*r* = −0.45 to −0.53, all *p* ≤ 0.03). We confirm in a longitudinal setting that the age-related decline in K_glc_ is directly associated with declining performance on some tests of cognition but does not significantly affect Kacac.

## Introduction

The cerebral metabolic rate of glucose (CMR_glc_), measured using positron emission tomography (PET) with [^18^F]-fluoro-2-deoxyglucose (^18^F-FDG), is lower in cognitively normal older adults (Petit-Taboué et al., 1998; Kalpouzos et al., 2009; Nugent et al., 2014b). Quantifying the metabolic decline of the brain as it ages is important in order to better understand not only the relationship between aging-related cognitive and metabolic changes in the brain but also the difference between normal and pathological brain aging (Cunnane et al., 2016c). Indeed, brain glucose hypometabolism is well-known to be present in Alzheimer’s disease (AD) itself (Herholz, 1995, 2014) and in conditions associated with increased risk of AD including mild cognitive impairment (MCI; Albin et al., 2010; Pagani et al., 2015), carriers of the apolipoprotein E4 (APOE4) allele (Mosconi et al., 2008a), a family history of AD (Reiman et al., 2004; Mosconi et al., 2007a), and type 2 diabetes (Baker et al., 2011).

Brain glucose hypometabolism appears to increase the risk of AD (Cunnane et al., 2011; Mosconi, 2013), but few longitudinal follow-up studies on brain energy metabolism during cognitively normal aging have been reported. Of those few reports (Mosconi et al., 2008b; Shokouhi et al., 2013), none have quantitatively evaluated metabolism of both glucose and ketones, the fat-derived alternative fuel for the brain when glucose is limiting. Ketone metabolism can be assessed by PET with the tracer—[^11^C]-acetoacetate (^11^C-AcAc). The assessment of both the brain’s main fuels is particularly informative given that brain ketone uptake remains normal whereas brain glucose hypometabolism is observed in MCI and early AD (Cunnane et al., 2016b; Croteau et al., 2018).

Our overall aim here was to conduct a longitudinal study in a group of cognitively normal older adults, focussing particularly on brain metabolism of both glucose and ketone in relation to cognitive function. Our primary objective was to quantify brain energy metabolism at 2-year intervals over 4 years, with a focus on the brain’s capacity to extract these fuels from the blood, defined as the rate constant for glucose (K_glc_; min^−1^) or acetoacetate (K_acac_; min^−1^). Our secondary aims were to assess brain structural changes and the relation between change in cognitive performance and changes in brain energy metabolism, brain structure, peripheral energy metabolism and body composition over the same 4-year period.

## Materials and Methods

### Participants

This study was approved by the ethic committee of the Research Center on Aging—CIUSSS de l’Estrie—CHUS, and was conducted with the informed written consent of all the participants. Of 41 participants in a previous cross-sectional study (Nugent et al., 2014b, 2016), 25 agreed to be followed-up. Exclusion criteria at the entry of the study included a Mini-Mental State Examination (MMSE) score <26/30, prescription drug addiction, alcohol abuse, depression, smoking, diabetes, overt evidence of heart, liver or renal disease, uncontrolled hypertension, dyslipidemia, or thyroid disease. Twelve of the 25 older adult participants were unmedicated; thirteen were taking prescription medication for hypertension (*N* = 8), elevated cholesterol (*N* = 4), or hypothyroidism (*N* = 4). At baseline (*N* = 25), and at the 2-year (*N* = 25) and 4-year (*N* = 16) follow-up, participants underwent a general medical examination, complete blood test, body composition analysis, cognitive evaluation and a PET-MRI protocol all within a 6-week period.

### PET Image Acquisition

Measurement of brain ^18^F-FDG and ^11^C-AcAc uptake was based on our dynamic quantitative PET imaging protocol described previously (Nugent et al., 2014b; Castellano et al., 2015b; Courchesne-Loyer et al., 2017). Brain PET scans were performed on a Philips Gemini TF PET/CT scanner (Philips Medical System, Eindhoven, Netherlands) using a dynamic list mode acquisition, with time-of-flight enabled, an isotropic voxel size of 2 mm^3^, field-of-view of 25 cm, and an axial field of 18 cm. Briefly, for each scan, after a fasting period of 6–7 h after breakfast, the participant was positioned in the PET/CT scanner in the early afternoon under dim light in a quiet environment, 15 min prior to acquiring the scan. Immediately after intravenous administration of 287 ± 90 MBq of ^11^C-AcAc *via* a forearm vein catheter, dynamic scans were obtained over a total duration of 10 min (time frames 12 × 10 s, 8 × 30 s, and 1 × 4 min). After a 60 min wash-out period, an i.v. dose of 191 ± 25 MBq of ^18^F-FDG was administered and PET images were acquired over 60 min (time frames = 12 × 10 s, 8 × 30 s, 6 × 4 min, and 6 × 5 min). Repeated arterialize venous blood samples were obtained during the two scans. ^18^F-FDG PET scans were reviewed by a nuclear radiologist to detect abnormalities.

### MR Image Acquisition

For all participants and at each time point, T1-weighted (T1w) and FLAIR MRIs were acquired on a 1.5 Tesla scanner (Sonata, Siemens Medical Solutions, Erlangen, Germany). Scan duration was 9.14 min. FLAIR and T1w MRIs were reviewed by a neurologist to detect structural abnormalities as well as potential ischemic stroke. Acquisition parameters were identical at all three time points (Nugent et al., 2014a):

- T1w: TR = 16.00 ms, TE = 4.68 ms, field of view = 256 × 240 × 192 mm, matrix size of 256 × 256 × 164, flip angle = 20° and 1 mm^3^ isotropic voxels; and
- FLAIR: TR = 8,500 ms, TE = 91 ms, TI = 2,400 ms, echo train length of 17, matrix size of 256 × 192, for a 230 × 172.5 mm^2^ field of view, slice thickness of 6 mm, spacing between slices of 1.2 mm.

### Quantification of Brain Acetoacetate, Total Ketone and Glucose Consumption

Brain ^11^C-AcAc and ^18^F-FDG PET images were analyzed using PMOD 3.8 software (PMOD Technologies Ltd., Zurich, Switzerland) as previously described (Castellano et al., 2017). Briefly, cerebral metabolic rate [CMR; (μmoles/100 g/min)] of acetoacetate and glucose (CMR_acac_ and CMR_glc_, respectively) were quantified according to the graphical analysis method developed by Patlak et al. (1983); based on the plasma time-activity curves calibrated against the series of blood samples obtained during the PET scans. For each tracer, the measured PET activity is divided by plasma activity, and plotted at a “normalized time” (integral of input curve from injection divided by plasma activity). The resulting slope is the rate constant for brain extraction of the tracer [K; (min^−1^)]. Subsequently, the CMR is derived from the brain influx K according to the following mathematical equation:

(1) CMR = (K × Cp)/LC

where Cp is the plasma tracer, and LC is the lumped constant. The LC of CMR_acac_ and CMR_glc_ were set to 1.0 and 0.8, respectively.

PET images were partial volume-corrected (PVC) using the PVC MR-based solution (Müller-Gärtner et al., 1992) implemented in PMOD (PMOD Technologies Ltd., Zurich, Switzerland). Brain segmentation was defined by T1w MRI automatic anatomical labeling (AAL Single-Subject Atlas) implemented in PNEURO tool (PMOD Technologies Ltd., Zurich, Switzerland). Brain 3D surface projections of voxel-wise parametric images of K_glc_ and K_acac_ obtained with PXMOD tool (PMOD Technologies Ltd., Zurich, Switzerland) were generated using the MIMvista medical program 6.4 (MIM Software Inc., Cleveland, OH, USA).

### Structural MR Image Analysis

T1w MRI data were automatically processed with the longitudinal stream in FreeSurfer (version 6.0^1^; Reuter et al., 2012). Regional brain volumes were normalized to intracranial volumes (Westman et al., 2013). A fully automated analysis of whole-brain atrophy was performed using the SIENA software package from version 4.1.4 of FSL (FMRIB’s Software Library^2^), from which the percent whole brain volume change (PBVC) was calculated at 2-year intervals (Smith et al., 2002). Global brain atrophy was expressed as a negative PBVC between two time points.

### Cognitive Battery

General cognitive status was based on the MMSE and Montreal Cognitive Assessment (MoCA). The Trail Making Number-Letter sequencing (NLS), Verbal Fluency category-switching and Stroop Color-Word interference inhibition/switching tests from the Delis–Kaplan Executive Function System (D-KEFS) provided information on executive function (Delis et al., 2001). Working memory was evaluated using the Number-Letter sequencing and the Spatial Span Backward tests of the Wechsler Adult Intelligence Scale-III (WMS-III; Wechsler, 1997), as well as the Digit Span Backward and Sequencing from the Wechsler Adult Intelligence Scale-IV (WAIS-IV; Wechsler, 2008). Episodic memory was assessed with the Rey Complex Figure immediate and delay recall tests (RCFT; Meyers and Meyers, 1995) as well as Verbal Paired Associates and Logical Memory recall and delay recall subtests from the WMS-III (Wechsler, 1997). The Boston Naming test, revised version (Kertesz, 1982), was used to provide information on language. Processing speed and attention were assessed with the Digit Symbol Substitution and the Symbol Search tests from the WAIS-IV as well as the Trail Making Visual Scanning (VS), Number Sequence (NS), and Letter Sequence (LS) tests from the D-KEFS (Wechsler, 2008). For each cognitive domain a composite z-score was calculated from the different scaled scores, based on normative conversion tables. For the 60-item Boston Naming test, normative data were used (Zec et al., 2007).

### Blood Metabolites and Body Composition

The plasma ketones, beta-hydroxybutyrate (BHB) and AcAc, were analyzed as previously described (St-Pierre et al., 2017). Plasma insulin was analyzed by ELISA (Alpco, Salem, NH, USA). An index of insulin resistance was derived using the homeostasis model assessment for insulin resistance (HOMA-IR) method (Matthews et al., 1985). All other blood assays were done at the biochemistry core laboratory of Sherbrooke University Hospital Center (CIUSSS de l’Estrie—CHUS, Sherbrooke, QC, USA). Plasma glucose, cholesterol and triglycerides were analyzed by automated colorimetric assay with commercially available kits on a clinical chemistry analyzer (Dimension Xpand Plus; Siemens, Deerfield, IL, USA). Plasma albumin, aspartate aminotransferase, alanine aminotransferase, creatinine, and high and low-density lipoprotein cholesterol were measured by commercially available kits on an automated analyzer (COBAS; Roche Diagnostics, Indianapolis, IN, USA). Glycated hemoglobin (HbA1c) was measured by high performance liquid chromatography (Tosoh Bioscience, King of Prussia, PA, USA). Thyroid-stimulating hormone (TSH) was measured by sandwich electro-chemiluminescence immunochemistry.

Measurement of body composition (fat and lean mass) was done by dual-energy X-ray absorptiometry (Lunar iDXA, GE Healthcare Lunar, Madison, WI, USA). DXA-derived measures, fat mass index (total fat mass/height^2^) and appendicular lean mass index (arm + leg lean mass/height^2^), were also calculated (Imboden et al., 2017a,b).

### Statistics

Results are expressed as mean values with their pooled standard errors, except where specified. The data were analyzed for serial measures using the MIXED procedure of SPSS (IBM SPSS Statistics Version 24.0) with age as the main factor. A linear model between dependent variables and age was used and slope tests for fixed effects were considered significant at an alpha level of 0.05. All neuroimaging analysis underwent a *p* ≤ 0.05 false discovery rate (FDR) correction (Benjamini and Hochberg, 1995). All correlations were made on the subject averages measurements. Linear regression modeling was used to test the different relationships: brain energy metabolism (CMR_glc_, CMR_acac_, K_glc_ and K_acac_) vs. cognition, brain morphometry vs. cognition, HOMA-IR vs. brain energy metabolism, and HOMA-IR vs. cognition.

## Results

### Brain Glucose and Acetoacetate Extraction

**Figure 1:**
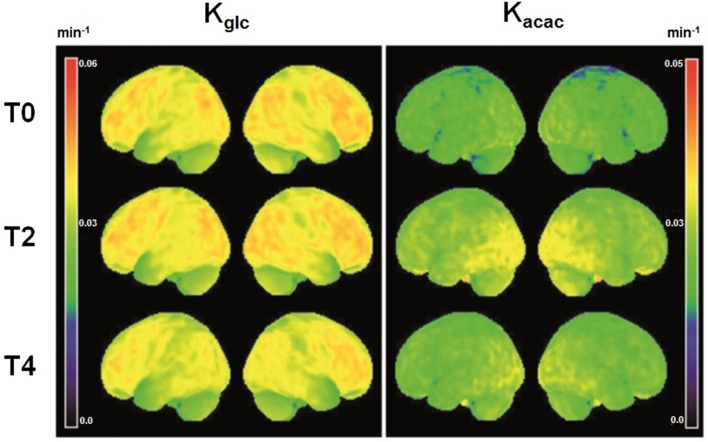
Voxel-wise three dimensional view of the brain surface showing the rate constant for brain extraction of glucose (K_glc_, min^−1^) and acetoacetate (K_acac_, min^−1^) at baseline (T0, *N* = 25) and two (T2, *N* = 25) and 4 years later (T4, *N* = 16). Theses mean maps are a simple representation of the average quantitative value of brain fuel uptake (glucose or acetoacetate) of all participants for each voxel relative to total whole brain tracer uptake.

Participant characteristics are shown in Table 1. Among the 43 neuroanatomical regions extracted using the AAL atlas, many brain regions had a statistically significant decrease in K_glc_ over time, mainly in the tempo-parietal lobes and cingulate gyri (Figure 1 and Table 2; all *p* < 0.05 FDR corrected). In contrast, K_acac_ did not show any statistical difference across the regions during the 4-year follow-up (Table 3; all *p* ≥ 0.11 FDR-corrected). At the 2 and 4-year time points compared to baseline, there was no difference in regional CMR_glc_ or CMR_acac_ (Supplementary Tables S1, S2; all *p* > 0.06 FDR-corrected).

**Table 1:** Characteristics of the participants during the 4 year follow-up.

Parameters	T0	T2	T4	SEM	Fixed effects estimation	*p*-value
	Mean	Mean	Mean			
*N*	25	25	16			
Gender (M/F)	10/15	10/15	8/8			
Age (*y*)	70.9	73.1	74.9	0.6		
Body mass index (kg/m^2^)	27.7	27.0	27.3	0.6	−0.001	*0.999*
Glucose (mM)	5.1	5.2	5.1	0.1	+0.017	*0.224*
Insulin (UI/L)	7.1	3.8	4.5	0.7	−0.130	*0.434*
HOMA-IR	1.1	0.9	0.7	0.1	−0.023	*0.553*
Glycated hemoglobin (%)	5.8	5.7	5.7	0.1	+0.005	*0.681*
Beta-Hydroxybutyrate (μM)	240	320	250	30	+0.007	*0.284*
Acetoacetate (μM)	120	180	130	10	+0.004	*0.165*
ALT (UI/L)	20.6	19.0	17.8	0.7	−0.047	*0.742*
AST (UI/L)	23.2	21.5	22.6	0.5	+0.131	*0.275*
Albumin (g/L)	42.3	41.3	40.8	0.3	−0.029	*0.708*
Total cholesterol (mM)	5.0	4.7	4.4	0.2	−0.019	*0.605*
Triglycerides (mM)	1.20	1.14	1.32	0.1	+0.026	*0.194*
HDL cholesterol (mM)	1.5	1.4	1.5	0.1	−0.005	*0.620*
LDL cholesterol (mM)	3.0	2.7	2.2	0.1	−0.196	*0.512*
Creatinine (μM)	76	74	82	2	−0.169	*0.711*
TSH (mUI/L)	2.5	2.1	2.2	0.1	−0.026	*0.282*

*ALT, alanine aminotransferase; AST, aspartate aminotransferase; HOMA-IR, homeostasis model assessment of insulin resistance (Matthews et al., 1985); TSH, Thyroid-stimulating hormone*.

**Table 2:** Regional rate constant for brain extraction of glucose (K_glc_; min^−1^) during the 4 year follow-up.

Regions of interest	T0	T2	T4	SEM	Fixed effects estimation	*p*-value
	Mean	Mean	Mean			
Frontal lobe						
Precentral	0.057	0.054	0.054	0.001	−0.0006	*0.077*
Superior frontal	0.057	0.053	0.053	0.002	−0.0006	*0.084*
Orbital superior frontal	0.052	0.050	0.053	0.001	−0.0003	*0.921*
Middle frontal	0.061	0.057	0.056	0.002	−0.0006	*0.134*
Orbital frontal	0.058	0.054	0.056	0.002	−0.0003	*0.492*
Opercular Inferior frontal	0.056	0.053	0.054	0.002	−0.0004	*0.179*
Triangular Inferior frontal	0.058	0.055	0.055	0.002	−0.0005	*0.164*
Orbital Inferior frontal	0.054	0.050	0.051	0.002	−0.0005	*0.162*
Rolandic operculum	0.049	0.049	0.049	0.001	−0.0002	*0.510*
Supplementary motor area	0.054	0.050	0.050	0.001	−0.0006	*0.065*
Olfactory cortex	0.036	0.035	0.034	0.001	−0.0003	*0.216*
Medial superior frontal	0.054	0.050	0.049	0.001	−0.0006	*0.062*
Orbital superior frontal	0.053	0.049	0.048	0.001	−0.0007	0.031*
Gyrus rectus	0.049	0.046	0.047	0.001	−0.0003	*0.274*
Paracentral	0.049	0.048	0.048	0.001	−0.0003	*0.316*
Temporal lobe						
Hippocampus	0.027	0.026	0.025	0.001	−0.0003	**0.050**
Parahippocampus	0.033	0.032	0.031	0.001	−0.0004	**0.048**
Amygdala	0.027	0.025	0.024	0.001	−0.0004	0.011*
Fusiform gyrus	0.042	0.040	0.039	0.001	−0.0004	*0.127*
Heschl gyrus	0.061	0.058	0.058	0.002	−0.0005	*0.144*
Superior temporal	0.050	0.047	0.047	0.002	−0.0005	*0.117*
Temporal pole	0.041	0.037	0.036	0.001	−0.0007	0.016*
Middle temporal	0.050	0.047	0.046	0.002	−0.0005	*0.109*
Inferior temporal	0.045	0.043	0.043	0.001	−0.0002	*0.395*
Parietal lobe						
Postcentral	0.054	0.051	0.051	0.002	−0.0005	*0.139*
Superior parietal	0.055	0.052	0.051	0.001	−0.0005	*0.102*
Inferior parietal	0.059	0.055	0.053	0.002	−0.0007	0.042*
Supramarginal	0.054	0.051	0.050	0.002	−0.0007	*0.057*
Angular	0.057	0.053	0.052	0.002	−0.0007	*0.052*
Precuneus	0.058	0.055	0.054	0.002	−0.0007	0.044*
Occipital lobe						
Calcarine	0.054	0.051	0.050	0.001	−0.0004	0.046*
Cuneus	0.058	0.052	0.055	0.002	−0.0007	*0.075*
Lingual	0.048	0.046	0.046	0.001	−0.0004	*0.207*
Superior occipital	0.055	0.051	0.051	0.002	−0.0007	0.038*
Middle occipital	0.053	0.049	0.049	0.002	−0.0006	*0.094*
Inferior occipital	0.049	0.048	0.048	0.002	−0.0002	*0.576*
Insula and cingulate gyri						
Insula	0.045	0.044	0.044	0.001	−0.0003	*0.280*
Anterior cingulate	0.045	0.041	0.040	0.001	−0.0006	0.016*
Middle cingulate	0.052	0.049	0.047	0.001	−0.0008	0.003*
Posterior cingulate	0.055	0.053	0.051	0.001	−0.0006	*0.054*
Central structures						
Caudate	0.040	0.039	0.038	0.001	−0.0002	*0.315*
Putamen	0.042	0.041	0.042	0.001	−0.0002	*0.330*
Thalamus	0.038	0.038	0.036	0.001	−0.0002	*0.199*

P-value significant indicated in bold (p ≤ 0.05); *Statistically significant after false discovery rate (FDR) correction (p < 0.05).

**Table 3:** Regional rate constant for brain extraction of acetoacetate (K_acac_; min^−1^) over the 4 year follow-up.

Regions of interest	T0	T2	T4	SEM	Fixed effects estimation	*p*-value
	Mean	Mean	Mean			
Frontal lobe						
Precentral	0.026	0.026	0.025	0.003	+0.0001	*0.594*
Superior frontal	0.025	0.025	0.024	0.003	+0.0001	*0.725*
Orbital superior frontal	0.025	0.025	0.024	0.003	+0.0003	*0.156*
Middle frontal	0.028	0.027	0.025	0.003	+0.0001	*0.614*
Orbital frontal	0.028	0.027	0.025	0.003	+0.0002	*0.392*
Opercular Inferior frontal	0.026	0.025	0.024	0.003	+0.0001	*0.680*
Triangular Inferior frontal	0.027	0.026	0.024	0.003	+0.0001	*0.624*
Orbital Inferior frontal	0.026	0.025	0.024	0.003	+0.0001	*0.478*
Rolandic operculum	0.024	0.023	0.022	0.003	+0.0001	*0.593*
Supplementary motor area	0.026	0.026	0.025	0.003	+0.0001	*0.505*
Olfactory cortex	0.019	0.018	0.017	0.002	+0.0001	*0.815*
Medial superior frontal	0.026	0.025	0.023	0.003	+0.0001	*0.711*
Orbital superior frontal	0.025	0.024	0.022	0.003	+0.0001	*0.616*
Gyrus rectus	0.024	0.024	0.022	0.003	+0.0001	*0.593*
Paracentral	0.029	0.029	0.028	0.004	+0.0002	*0.373*
Temporal lobe						
Hippocampus	0.019	0.018	0.017	0.002	+0.0001	*0.678*
Parahippocampus	0.021	0.021	0.019	0.003	+0.0001	*0.907*
Amygdala	0.019	0.016	0.016	0.002	+0.0001	*0.807*
Fusiform gyrus	0.025	0.024	0.023	0.003	+0.0001	*0.648*
Heschl gyrus	0.026	0.024	0.024	0.003	+0.0002	*0.665*
Superior temporal	0.026	0.025	0.024	0.003	+0.0001	*0.567*
Temporal pole	0.023	0.022	0.020	0.003	+0.0002	*0.883*
Middle temporal	0.028	0.027	0.026	0.003	+0.0001	*0.552*
Inferior temporal	0.028	0.026	0.025	0.003	+0.0001	*0.567*
Parietal lobe						
Postcentral	0.027	0.027	0.025	0.003	+0.0001	*0.477*
Superior parietal	0.030	0.029	0.028	0.004	+0.0002	*0.409*
Inferior parietal	0.029	0.028	0.027	0.003	+0.0002	*0.457*
Supramarginal	0.028	0.027	0.026	0.003	+0.0001	*0.524*
Angular	0.029	0.028	0.027	0.004	+0.0001	*0.636*
Precuneus	0.030	0.029	0.028	0.004	+0.0002	*0.390*
Occipital lobe						
Calcarine	0.030	0.029	0.028	0.004	+0.0002	*0.347*
Cuneus	0.032	0.031	0.031	0.004	+0.0003	*0.283*
Lingual	0.027	0.027	0.026	0.003	+0.0003	*0.194*
Superior occipital	0.030	0.030	0.029	0.004	+0.0002	*0.332*
Middle occipital	0.030	0.030	0.029	0.004	+0.0002	*0.329*
Inferior occipital	0.030	0.030	0.029	0.004	+0.0004	*0.110*
Insula and cingulate gyri						
Insula	0.022	0.021	0.020	0.003	+0.0001	*0.924*
Anterior cingulate	0.022	0.021	0.019	0.003	+0.0001	*0.986*
Middle cingulate	0.026	0.025	0.024	0.003	+0.0001	*0.590*
Posterior cingulate	0.028	0.026	0.025	0.003	+0.0001	*0.530*
Central structures						
Caudate	0.016	0.016	0.015	0.002	+0.0001	*0.841*
Putamen	0.019	0.018	0.017	0.002	+0.0001	*0.970*
Thalamus	0.021	0.020	0.019	0.003	+0.0001	*0.704*

P-value significant indicated in bold (p ≤ 0.05); *Statistically significant after FDR correction (p < 0.05).

### Global and Regional MRI Measures

During the 2 and 4-year follow-ups, global brain atrophy (mean ± SD) was 1.2 ± 1.2% and 1.7 ± 1.7%, respectively, or 0.52% annually. Regional analysis of brain volumes showed the same pattern as global brain atrophy (Supplementary Table S3). Cortical thickness decreased by a mean of 0.04 ± 0.01 mm/year, mainly in the fronto-temporo-parietal regions (Supplementary Table S4; all *p* ≤ 0.01 FDR-corrected).

### Cognitive Evaluation

Participants had a mean of 16 ± 4 years education. Overall, cognitive performance at baseline and over the 4-year follow-up remained within the normal range for age (Table 4), but significant declines in raw scores were noted on certain tests. For instance, time to complete increased on the Trail Making VS, NS, LS and NLS tasks and Stroop Inhibition/Switching task (all *p* ≤ 0.05). On the other hand, performance on learning word pairs of the Verbal Paired Associates—Part I (VPAI) improved over time (*p* = 0.03). After normalization of the results for age (scaled scores), only differences on the Trail Making VS and VPAI tests remained statistically different (Estimated fixed effects = −0.148 and +0.292, respectively; all *p* ≤ 0.02). Composite z-scores did not show any significant change over the 4 years, except in language which showed improvement (z-score ± SD, from −0.6 ± 1.1 to −0.1 ± 0.9; *p* = 0.02).

**Table 4:** Cognitive test scores (mean ± SEM) during the 4 year follow-up.

Cognitive tests	T0	T2	T4	SEM	Fixed effects estimation	*p*-value
	Mean	Mean	Mean			
General cognition						
MMSE (score/30)	29.3	29.3	29.3	0.1	−0.027	*0.300*
MOCA (score/30)	26.6	26.9	26.3	0.3	−0.071	*0.314*
Executive function						
D-KEFS Stroop Inhibition/Switching (s)	74.5	75.5	81.7	3.4	+1.559	**0.026**
D-KEFS Verbal Fluency Category Switching (total correct responses)	13.4	12.9	12.5	0.3	−0.080	*0.295*
D-KEFS Trail Number-Letter Sequence (s)	100.6	98.6	109.2	6.3	+2.144	*0.053*
*Composite z-score*	*+0.3*	*+0.4*	*+0.4*	*0.1*	*+0.001*	*0.968*
Working memory						
WMS-III Letter-Number Sequencing (total raw score/21)	10.1	9.8	9.9	0.3	−0.072	*0.212*
WMS-III Spatial Span Backward (raw score/16)	6.7	6.6	6.6	0.2	−0.063	*0.237*
WAIS-IV Digit Span Backward (raw score/19)	7.8	7.4	7.6	0.3	−0.007	*0.912*
WAIS-IV Digit Span Sequencing (raw score/19)	7.7	8.1	8.1	0.3	−0.065	*0.313*
*Composite z-score*	*+0.3*	*+0.3*	*+0.4*	*0.1*	*+0.027*	*0.172*
Episodic memory (immediate + delay recall)						
Rey Complex Figure Test—Immediate Recall (total raw score/36)	24.2	24.9	23.9	0.7	−0.146	*0.354*
Rey Complex Figure Test—Delay Recall (total raw score/36)	24.5	25.1	23.7	0.6	−0.163	*0.244*
WMS-III Verbal Paired Associated I Recall (total raw score/32)	21.9	23.2	21.6	0.9	+0.413	**0.026**
WMS-III Verbal Paired Associated II Delay Recall (total raw score/8)	6.6	6.8	6.3	0.2	−0.013	*0.818*
WMS-III Logical Memory I Recall (total raw score/75)	48.4	48.2	49.2	0.9	−0.063	*0.776*
WMS-III Logical Memory II Delay Recall (total raw score/50)	30.8	30.7	32.2	0.7	−0.039	*0.824*
*Composite z-score*	*+1.7*	*+1.8*	*+1.9*	*0.1*	*+0.034*	*0.111*
Language						
Boston Naming Test (total raw score/60)	52.1	52.8	52.8	0.6	+0.055	*0.609*
*Composite z-score*	−0.6	−0.4	−0.1	0.1	+0.064	**0.022**
Attention and processing speed						
WAIS-IV Symbol Search (total correct responses)	23.4	23.3	24.2	0.8	−0.177	*0.288*
WAIS-IV Code (total correct responses)	58	55.5	63.4	1.8	−0.217	*0.524*
D-KEFS Trail Making Visual Scanning (s)	23.9	22.9	24.0	0.9	+0.570	**0.002**
D-KEFS Trail Number Sequence (s)	40.2	41.4	38.9	1.9	+1.316	**0.001**
D-KEFS Trail Letter Sequence (s)	38.6	45.9	44.5	2.4	+1.128	**0.038**
*Composite z-score*	*+0.5*	*+0.5*	*+0.7*	*0.1*	*+0.006*	*0.714*

*P-value significant indicated in bold (p ≤ 0.05)*.

### Blood Parameters and Body Composition

Baseline anthropometry and plasma metabolites corresponded to normal reference values for the same age range from the Sherbrooke University Hospital Center (Sherbrooke, QC, USA). Over the 4-year follow-up, no significant change in plasma metabolites or HOMA-IR index was observed (Table 1; all *p* ≥ 0.19). Participants had an average BMI of 27 ± 0.7 kg/m^2^ throughout the 4 years. No significant difference was found in body fat parameters throughout the 4 years (data not shown; all *p* ≥ 0.11). However, appendicular lean mass index declined over the 4 years (baseline, 2-year and 4-year values of 8.1 ± 1.2, 7.9 ± 1.2 and 7.8 ± 1.3 kg/m^2^, respectively; all *p* ≤ 0.02) but remained within the normal range for age (Imboden et al., 2017a).

### Relation Between Brain Function/Structure, Cognitive Performance and Insulin Resistance

A significant positive relationship was observed between higher K_glc_ in the caudate and higher score on both the WAIS-IV Digit span Backward (*r* = +0.41, *p* = 0.044) and the D-KEFS Trail Making Visual scanning tests (*r* = +0.43, *p* = 0.034). Similarly, higher K_acac_ in the caudate was associated with a higher performance on several components of the D-KEFS Stroop Color-Word, the D-KEFS Verbal Fluency and the WMS-III Verbal Paired Associate (Supplementary Table S5; all *p* ≤ 0.04). Higher HOMA-IR was associated with lower K_glc_ in the thalamus (*r* = −0.44, *p* = 0.04) and caudate (*r* = −0.43; *p* = 0.05). Higher HOMA-IR tended to be associated with lower plasma ketone levels (*r* = −0.40, *p* = 0.06), but no significant relationship was noted between HOMA-IR and regional K_acac_ (*p* ≥ 0.51). Lower composite z-scores for executive function (*r* = −0.45, *p* = 0.035), attention and processing speed (β = −0.53, *p* = 0.012), were significantly inversely related to HOMA-IR. Better cognitive performance on several tests was positively associated with higher volume of the caudate (Rey Complex Figure Test—Delay Recall scaled score: *r* = +0.50, *p* = 0.012; Logical Memory Total scaled score: *r* = +0.41, *p* = 0.048; WAIS-IV Digit Span Backward scaled score: *r* = +0.56, *p* = 0.004). No other significant relationship was observed among these different parameters (all *p* > 0.05).

## Discussion

Our main observation is that over the 4 year follow-up, cognitively normal older people have a significant decline in K_glc_, the brain’s capacity to extract or acquire glucose, in multiple brain regions. This change in K_glc_ over time was associated with declining cognitive performance which nevertheless remained within the normal range for age. Several brain regions (frontal, parietal and temporal lobes) demonstrated an annual decline in K_glc_ averaging 1.5%–3.0% per year. These aging-related changes in brain glucose metabolism were less severe, less extensive and in different regions from the rate of decline in K_glc_ in the frontal and temporal regions in AD (Jagust et al., 1991; Piert et al., 1996; Kondoh et al., 1997; Mosconi et al., 2007b; Castellano et al., 2015b). No such temporal change occurred in the brain’s capacity to acquire acetoacetate (K_acac_). Hence, we show for the first time in a longitudinal setting that the aging-related decline in brain energy metabolism is specific to glucose and is a physiological change associated with cognitive changes that are considered normal for age.

In the present study, we have focussed on “K,” K_glc_ or K_acac_ because it is a direct measure of the brain’s capacity to take up these to energy substrates. CMR is the brain’s actual uptake of the tracer which varies with changes in blood glucose or ketones. Plasma glucose does not vary too much in this type of PET study, so CMR_glc_ is usually tightly correlated to K_glc_ and the two measures show broadly the same differences across the brain or over time. However, because plasma ketones can vary a lot, whereas K_acac_ does not vary much, K_acac_ is a better measure of the brain’s actual capacity to obtain ketones when they are available.

Decreasing brain glucose metabolism as function of age has been reported in rats (Smith et al., 1980), dogs (London et al., 1983), macaques (Noda et al., 2003) and humans (Bentourkia et al., 2000; Kalpouzos et al., 2009; Chételat et al., 2013; Nugent et al., 2014a,b, 2016). Several cross-sectional ^18^F-FDG PET studies, mostly done by comparing young vs. older healthy adults, have reported that age-related differences in brain energy metabolism mainly affect the frontal and temporo-parietal lobes (see review by Berti et al., 2014). However, few longitudinal studies on brain energy metabolism during aging have been reported. One 4-year follow-up reported subtle regional metabolic changes in the brain associated with changes in cognition in healthy older adults (Shokouhi et al., 2013). A 24-month follow-up study in older individuals with subjective memory complaints recently reported a reduced standardized uptake value for ^18^F-FDG in the posterior cingulate and temporo-parietal brain regions, a difference which disappeared after adjustment for sex, age and education (Dubois et al., 2018). Ours is the first report to quantitatively compare both brain glucose and AcAc metabolism in a longitudinal follow-up with measurements at three time points over 4 years.

The rate of decline in whole brain volume of 0.52%/year in the present study was comparable to that reported in other longitudinal studies in a healthy older population (Enzinger et al., 2005; Jack et al., 2005; Leong et al., 2017). Our participants had decreasing gray matter volume in most brain regions, including sub-cortical nuclei such as the caudate and putamen. There was also a significant correlation between the volume of the caudate and performance on learning tasks. The role of the caudate in cognitive decline has been previously noted (Grahn et al., 2008). Similar to our results, Bauer et al. (2015) reported that atrophy of the caudate is correlated with declining associative learning during normal aging. We extend these reports with a direct and significant relationship between lower performance in several cognitive tests of attention and executive function and lower K_glc_ in the caudate.

Mild to moderate systemic insulin resistance is common in aging (Rowe et al., 1983), so we investigated the potential relation between the HOMA-IR as a common measure of insulin resistance and brain energy metabolism. Similar to other studies that reported a negative association between insulin resistance and CMR_glc_ (Baker et al., 2011; Castellano et al., 2015a; Willette et al., 2015), we show here that a higher HOMA-IR correlated significantly with lower K_glc_ and tended to correlate with lower plasma ketones (*p* = 0.06). Insulin influences three aspects of ketone metabolism: free fatty acid availability from adipose tissue by lipolysis, ketogenesis in the liver and peripheral ketone clearance (Ciaraldi and Henry, 2004). Ketone transport into tissues is influenced by insulin (Balasse and Havel, 1971; Hall et al., 1984) and the inhibitory influence of insulin resistance on ketone body metabolism can be present at raised but still physiological insulin levels (Singh et al., 1993). In the present study, HOMA-IR was inversely related to composite z-score for executive function, attention and processing speed, results that agree with those reported in older adults with cardiovascular disease (Lutski et al., 2017). The present results go further in demonstrating the direct relationship between cognition and the central utilization of glucose, and that insulin resistance is associated with lower plasma ketones in later life. This observation may be important in ketogenic strategies designed to compensate for or bypass brain glucose hypometabolism in AD (Cunnane et al., 2016a).

This study had several limitations, the main one being the small and unbalanced sample size between baseline and year 2 (both *N* = 25) compared to year 4 (*N* = 16). However, our statistical approach based on the linear mixed-effects model is the one specifically recommended for an unequal number of observations (McCulloch et al., 2008). It would have been interesting to explore a possible gender difference since a disparity in cognition can be observed between older men and women (Munro et al., 2012). However, this comparison was not done owing to small sample size, which was the same reason apolipoprotein E genotype was not assessed. APOE4 is both an important risk factor for late onset AD and lower brain glucose metabolism is present in AD-vulnerable brain regions in cognitively normal middle-aged carriers of APOE4 (Reiman et al., 2001). Longitudinal changes in cognitive performance show heterogeneous trajectories in normal aging (Wilson et al., 2002; Goh et al., 2012). The relatively slow decline in cognition in the present study likely reflects the overall good health and education, as well as the relatively young age of our cohort (mid-70s at the end of the study). Decline on several domains of cognition including memory may not manifest until after 75 years of age (Small et al., 2011). Finally, since the participants in the present study had a cognitive performance within the normal range at all three time points, the metabolic changes we report here do not really help identify those at highest risk of progressing to MCI or AD.

## Conclusion

Widespread longitudinal changes in the capacity of the brain to extract glucose occur in healthy, cognitively normal older adults. These changes in brain glucose metabolism exceeded by several fold the annual rate of change in brain volume and were not observed for brain ketone metabolism. Insulin resistance was associated not only with declining cognitive performance but also with lower brain glucose metabolism in this cohort.

## Data Availability

All datasets generated for this study are included in the manuscript and the supplementary files.

## Author Contributions

SC designed the study. SC, MF and C-AC wrote the protocol. C-AC, EC, SN, ML and ST participated in the PET and/or MR image acquisition and processing. SD and OP performed the longitudinal MRI analysis. C-AC and CH ran the cognitive assessments, and analyzed and interpreted the cognitive data. ID supervised the iDXA acquisition and analysis. MF, VS-P and CV contributed to experimental methodology and biological analyses. CB, NF, TF and ET provided medical supervision and assessments throughout the study. C-AC undertook the statistical analysis. C-AC and SC drafted and revised the manuscript. All co-authors reviewed and commented on the manuscript before submission.

## Conflict of Interest Statement

SC has received travel honoraria and/or consulted for Accera, Bulletproof, Nisshin Oillio, and Prüvit; he has received test materials for research purposes and/or research funding awarded to the Université de Sherbrooke from Abitec, Ultragenyx and Nestlé. He has a patent application pending on the formulation of a ketogenic drink. SC is President and sole shareholder of Senotec Ltd., company aiming to develop a ketogenic supplement for human consumption. SD is officer and shareholder of True Positive Medical Devices Inc., a company specializing in brain image analysis services. The remaining authors declare that the research was conducted in the absence of any commercial or financial relationships that could be construed as a potential conflict of interest.

## Footnotes

^1^http://surfer.nmr.mgh.harvard.edu

^2^www.fmrib.ox.ac.uk/fsl

## Abbreviations

^11^C-AcAc: [^11^C]-acetoacetate
^18^F-FDG: [^18^F]-Fluoro-2-deoxyglucose
AD: Alzheimer’s disease
APOE4: apolipoprotein E4
BHB: Beta-Hydroxybutyrate
CMR_acac_: cerebral metabolic rate of acetoacetate
CMR_glc_: cerebral metabolic rate of glucose
HOMA-IR: Homeostasis Model Assessment of insulin resistance
K_acac_: rate constant for brain extraction of acetoacetate
K_glc_: rate constant for brain extraction of glucose
MCI: mild cognitive impairment
MMSE: Mini-mental state examination
MoCA: Montreal Cognitive Assessment
MRI: magnetic resonance imaging
PET: positron emission tomography.
