# Supplementary material for: Links Between Metabolic and Structural Changes in the Brain of Cognitively Normal Older Adults: A 4-Year Longitudinal Follow-Up
Source: Front Aging Neurosci. 2019 Jan 15;11:15. doi: 10.3389/fnagi.2019.00015 (PMC6384269; doi:10.3389/fnagi.2019.00015)
Supplement: Supplementary file 1 [file Table_1.docx]

Supplementary Table 1: Regional cerebral metabolic rate of glucose (CMR_glc_; μmol/100 g/min) during the four year follow-up.

| **Regions of interest** | |  | **T0** |  | **T2** |  | **T4** |  | **SEM** |  | ***Fixed Effects Estimation*** | ***p-value*** |
| --- | --- | --- | --- | --- | --- | --- | --- | --- | --- | --- | --- | --- |
|  |  |  | **Mean** |  | **Mean** |  | **Mean** |  |  |  |  |  |
| Frontal Lobe | |  |  |  |  |  |  |  |  |  |  |  |
|  | Precentral |  | 34.0 |  | 33.2 |  | 32.2 |  | 0.7 |  | -0.266 | *0.108* |
|  | Superior frontal |  | 34.3 |  | 32.8 |  | 31.8 |  | 0.8 |  | -0.266 | *0.129* |
|  | Orbital superior frontal |  | 31.2 |  | 31.2 |  | 31.6 |  | 0.8 |  | +0.082 | *0.662* |
|  | Middle frontal |  | 36.4 |  | 35.1 |  | 33.7 |  | 0.8 |  | -0.232 | *0.228* |
|  | Orbital frontal |  | 34.9 |  | 33.5 |  | 33.4 |  | 0.9 |  | -0.052 | *0.812* |
|  | Opercular Inferior frontal |  | 33.5 |  | 33.1 |  | 32.5 |  | 0.8 |  | -0.182 | *0.295* |
|  | Triangular Inferior frontal |  | 35.0 |  | 34.0 |  | 33.1 |  | 0.8 |  | -0.207 | *0.276* |
|  | Orbital Inferior frontal |  | 32.3 |  | 31.3 |  | 30.5 |  | 0.8 |  | -0.193 | *0.286* |
|  | Rolandic operculum |  | 29.5 |  | 30.1 |  | 29.5 |  | 0.7 |  | -0.046 | *0.780* |
|  | Supplementary motor area |  | 32.1 |  | 31.1 |  | 30.2 |  | 0.7 |  | -0.259 | *0.094* |
|  | Olfactory cortex |  | 21.7 |  | 21.7 |  | 20.3 |  | 0.5 |  | -0.106 | *0.401* |
|  | Medial superior frontal |  | 32.3 |  | 30.8 |  | 29.4 |  | 0.7 |  | -0.275 | *0.102* |
|  | Orbital superior frontal |  | 32.1 |  | 30.3 |  | 29.0 |  | 0.7 |  | -0.298 | *0.077* |
|  | Gyrus rectus |  | 29.3 |  | 28.7 |  | 28.1 |  | 0.6 |  | -0.099 | *0.513* |
|  | Paracentral |  | 29.7 |  | 29.9 |  | 29.0 |  | 0.6 |  | -0.058 | *0.697* |
| Temporal Lobe | |  |  |  |  |  |  |  |  |  |  |  |
|  | Hippocampus |  | 16.4 |  | 16.4 |  | 15.3 |  | 0.3 |  | -0.098 | *0.212* |
|  | Parahippocampus |  | 20.1 |  | 20.1 |  | 18.8 |  | 0.4 |  | -0.141 | *0.162* |
|  | Amygdala |  | 16.5 |  | 15.3 |  | 14.5 |  | 0.3 |  | -0.151 | *0.067* |
|  | Fusiform gyrus |  | 25.1 |  | 24.9 |  | 23.7 |  | 0.6 |  | -0.157 | *0.240* |
|  | Heschl gyrus |  | 36.6 |  | 36.0 |  | 34.9 |  | 0.8 |  | -0.198 | *0.262* |
|  | Superior temporal |  | 30.2 |  | 29.2 |  | 28.2 |  | 0.8 |  | -0.245 | *0.164* |
|  | Temporal pole |  | 24.6 |  | 22.6 |  | 21.3 |  | 0.7 |  | -0.373 | ***0.017*** |
|  | Middle temporal |  | 29.9 |  | 29.1 |  | 27.9 |  | 0.8 |  | -0.250 | *0.184* |
|  | Inferior temporal |  | 26.8 |  | 26.9 |  | 26.1 |  | 0.7 |  | -0.062 | *0.678* |
| Parietal Lobe | |  |  |  |  |  |  |  |  |  |  |  |
|  | Postcentral |  | 32.1 |  | 31.4 |  | 30.7 |  | 0.8 |  | -0.215 | *0.234* |
|  | Superior parietal |  | 32.8 |  | 32.4 |  | 30.8 |  | 0.7 |  | -0.178 | *0.305* |
|  | Inferior parietal |  | 35.5 |  | 33.8 |  | 32.0 |  | 0.9 |  | -0.346 | *0.103* |
|  | Supramarginal |  | 32.4 |  | 31.4 |  | 29.8 |  | 0.8 |  | -0.313 | *0.107* |
|  | Angular |  | 34.1 |  | 32.7 |  | 31.2 |  | 0.9 |  | -0.336 | *0.092* |
|  | Precuneus |  | 34.9 |  | 34.2 |  | 32.5 |  | 0.8 |  | -0.265 | *0.168* |
| Occipital Lobe | |  |  |  |  |  |  |  |  |  |  |  |
|  | Calcarine |  | 32.2 |  | 31.5 |  | 30.1 |  | 0.7 |  | -0.275 | *0.104* |
|  | Cuneus |  | 34.7 |  | 32.5 |  | 32.8 |  | 0.8 |  | -0.291 | *0.135* |
|  | Lingual |  | 28.6 |  | 28.4 |  | 27.6 |  | 0.6 |  | -0.107 | *0.480* |
|  | Superior occipital |  | 32.9 |  | 31.4 |  | 30.6 |  | 0.9 |  | -0.321 | *0.109* |
|  | Middle occipital |  | 31.5 |  | 30.3 |  | 29.3 |  | 0.9 |  | -0.278 | *0.185* |
|  | Inferior occipital |  | 29.5 |  | 29.7 |  | 28.8 |  | 0.8 |  | -0.019 | *0.926* |
| Insula and Cingulate Gyri | |  |  |  |  |  |  |  |  |  |  |  |
|  | Insula |  | 27.1 |  | 27.2 |  | 26.5 |  | 0.6 |  | -0.078 | *0.578* |
|  | Anterior Cingulate |  | 27.0 |  | 25.7 |  | 24.1 |  | 0.5 |  | -0.279 | ***0.030*** |
|  | Middle Cingulate |  | 31.2 |  | 30.1 |  | 28.3 |  | 0.7 |  | -0.372 | ***0.013*** |
|  | Posterior cingulate |  | 32.9 |  | 32.8 |  | 30.5 |  | 0.8 |  | -0.253 | *0.158* |
| Central Structures | |  |  |  |  |  |  |  |  |  |  |  |
|  | Caudate |  | 23.9 |  | 24.0 |  | 23.2 |  | 0.5 |  | -0.037 | *0.741* |
|  | Putamen |  | 25.3 |  | 25.7 |  | 25.2 |  | 0.5 |  | -0.049 | *0.673* |
|  | Thalamus |  | 23.1 |  | 23.3 |  | 21.9 |  | 0.5 |  | -0.071 | *0.523* |

P-value significant indicated in bold (*p* ≤ 0.05); * statistically significant after FDR correction (*p*< 0.05).

Supplementary Table 2: Regional cerebral metabolic rate of acetoacetate (CMR_acac_; μmol/100 g/min) over the four year follow-up.

| **Regions of interest** | |  | **T0** |  | **T2** |  | **T4** |  | **SEM** |  | ***Fixed Effects Estimation*** | ***p-value*** |
| --- | --- | --- | --- | --- | --- | --- | --- | --- | --- | --- | --- | --- |
|  |  |  | **Mean** |  | **Mean** |  | **Mean** |  |  |  |  |  |
| Frontal Lobe | |  |  |  |  |  |  |  |  |  |  |  |
|  | Precentral |  | 0.34 |  | 0.46 |  | 0.30 |  | 0.05 |  | +0.013 | *0.135* |
|  | Superior frontal |  | 0.32 |  | 0.45 |  | 0.28 |  | 0.04 |  | +0.013 | *0.133* |
|  | Orbital superior frontal |  | 0.32 |  | 0.45 |  | 0.29 |  | 0.05 |  | +0.017 | *0.066* |
|  | Middle frontal |  | 0.35 |  | 0.48 |  | 0.31 |  | 0.05 |  | +0.015 | *0.107* |
|  | Orbital frontal |  | 0.35 |  | 0.49 |  | 0.31 |  | 0.05 |  | +0.017 | *0.080* |
|  | Opercular Inferior frontal |  | 0.33 |  | 0.44 |  | 0.28 |  | 0.04 |  | +0.013 | *0.145* |
|  | Triangular Inferior frontal |  | 0.34 |  | 0.46 |  | 0.29 |  | 0.05 |  | +0.014 | *0.113* |
|  | Orbital Inferior frontal |  | 0.33 |  | 0.45 |  | 0.28 |  | 0.05 |  | +0.015 | *0.089* |
|  | Rolandic operculum |  | 0.31 |  | 0.42 |  | 0.26 |  | 0.04 |  | +0.012 | *0.120* |
|  | Supplementary motor area |  | 0.34 |  | 0.47 |  | 0.30 |  | 0.05 |  | +0.014 | *0.114* |
|  | Olfactory cortex |  | 0.24 |  | 0.32 |  | 0.20 |  | 0.03 |  | +0.009 | *0.148* |
|  | Medial superior frontal |  | 0.32 |  | 0.44 |  | 0.28 |  | 0.04 |  | +0.014 | *0.106* |
|  | Orbital superior frontal |  | 0.32 |  | 0.42 |  | 0.27 |  | 0.04 |  | +0.013 | *0.115* |
|  | Gyrus rectus |  | 0.31 |  | 0.42 |  | 0.26 |  | 0.04 |  | +0.014 | *0.100* |
|  | Paracentral |  | 0.37 |  | 0.51 |  | 0.34 |  | 0.05 |  | +0.016 | *0.089* |
| Temporal Lobe | |  |  |  |  |  |  |  |  |  |  |  |
|  | Hippocampus |  | 0.24 |  | 0.31 |  | 0.20 |  | 0.03 |  | +0.007 | *0.222* |
|  | Parahippocampus |  | 0.27 |  | 0.36 |  | 0.24 |  | 0.04 |  | +0.010 | *0.160* |
|  | Amygdala |  | 0.23 |  | 0.29 |  | 0.19 |  | 0.03 |  | +0.007 | *0.211* |
|  | Fusiform gyrus |  | 0.32 |  | 0.43 |  | 0.28 |  | 0.04 |  | +0.014 | *0.108* |
|  | Heschl gyrus |  | 0.33 |  | 0.43 |  | 0.28 |  | 0.04 |  | +0.012 | *0.158* |
|  | Superior temporal |  | 0.33 |  | 0.44 |  | 0.29 |  | 0.04 |  | +0.013 | *0.125* |
|  | Middle temporal |  | 0.35 |  | 0.47 |  | 0.31 |  | 0.05 |  | +0.014 | *0.125* |
|  | Temporal pole |  | 0.32 |  | 0.43 |  | 0.27 |  | 0.04 |  | +0.011 | *0.161* |
|  | Inferior temporal |  | 0.35 |  | 0.47 |  | 0.30 |  | 0.05 |  | +0.015 | *0.980* |
| Parietal Lobe | |  |  |  |  |  |  |  |  |  |  |  |
|  | Postcentral |  | 0.34 |  | 0.47 |  | 0.30 |  | 0.05 |  | +0.014 | *0.120* |
|  | Superior parietal |  | 0.39 |  | 0.52 |  | 0.33 |  | 0.05 |  | +0.017 | *0.096* |
|  | Inferior parietal |  | 0.36 |  | 0.50 |  | 0.32 |  | 0.05 |  | +0.016 | *0.108* |
|  | Supramarginal |  | 0.35 |  | 0.48 |  | 0.31 |  | 0.05 |  | +0.014 | *0.117* |
|  | Angular |  | 0.38 |  | 0.50 |  | 0.33 |  | 0.05 |  | +0.015 | *0.130* |
|  | Precuneus |  | 0.38 |  | 0.51 |  | 0.33 |  | 0.05 |  | +0.017 | *0.930* |
| Occipital Lobe | |  |  |  |  |  |  |  |  |  |  |  |
|  | Calcarine |  | 0.38 |  | 0.51 |  | 0.34 |  | 0.05 |  | +0.016 | *0.102* |
|  | Cuneus |  | 0.41 |  | 0.56 |  | 0.37 |  | 0.06 |  | +0.018 | *0.089* |
|  | Lingual |  | 0.35 |  | 0.49 |  | 0.31 |  | 0.05 |  | +0.017 | *0.074* |
|  | Superior occipital |  | 0.38 |  | 0.53 |  | 0.34 |  | 0.05 |  | +0.016 | *0.115* |
|  | Middle occipital |  | 0.38 |  | 0.52 |  | 0.35 |  | 0.05 |  | +0.016 | *0.113* |
|  | Inferior occipital |  | 0.38 |  | 0.53 |  | 0.34 |  | 0.05 |  | +0.019 | *0.063* |
| Insula and Cingulate Gyri | |  |  |  |  |  |  |  |  |  |  |  |
|  | Insula |  | 0.29 |  | 0.37 |  | 0.24 |  | 0.04 |  | +0.010 | *0.163* |
|  | Anterior Cingulate |  | 0.28 |  | 0.37 |  | 0.22 |  | 0.04 |  | +0.010 | *0.159* |
|  | Middle Cingulate |  | 0.33 |  | 0.44 |  | 0.28 |  | 0.05 |  | +0.013 | *0.124* |
|  | Posterior cingulate |  | 0.36 |  | 0.47 |  | 0.31 |  | 0.05 |  | +0.016 | *0.085* |
| Central Structures | |  |  |  |  |  |  |  |  |  |  |  |
|  | Caudate |  | 0.20 |  | 0.27 |  | 0.18 |  | 0.03 |  | +0.007 | *0.171* |
|  | Putamen |  | 0.25 |  | 0.32 |  | 0.21 |  | 0.03 |  | +0.009 | *0.179* |
|  | Thalamus |  | 0.26 |  | 0.35 |  | 0.23 |  | 0.04 |  | +0.008 | *0.222* |

P-value significant indicated in bold (*p* ≤ 0.05); * statistically significant after FDR correction (*p*< 0.05).

Supplementary Table 3: Regional brain volumes (ml) over the four year follow-up.

| **Regions of interest** | |  | **T0** |  | **T2** |  | **T4** |  | **SEM** |  | ***Fixed Effects Estimation*** | ***p-value*** |
| --- | --- | --- | --- | --- | --- | --- | --- | --- | --- | --- | --- | --- |
|  |  |  | **Mean** |  | **Mean** |  | **Mean** |  |  |  |  |  |
| Frontal Lobe | |  |  |  |  |  |  |  |  |  |  |  |
|  | Precentral |  | 22.8 |  | 21.6 |  | 20.4 |  | 3.1 |  | -0.17 | ***0.037*** |
|  | Superior frontal |  | 47.4 |  | 45.5 |  | 43.9 |  | 4.8 |  | -0.25 | ***0.042*** |
|  | Caudal middle frontal |  | 12.5 |  | 11.7 |  | 11.0 |  | 2.3 |  | -0.09 | ***0.050*** |
|  | Rostral middle frontal |  | 21.4 |  | 20.5 |  | 20.0 |  | 2.0 |  | -0.22 | ***0.001****** |
|  | Pars Opercularis |  | 7.8 |  | 7.5 |  | 7.3 |  | 1.2 |  | -0.04 | *0.119* |
|  | Pars Orbitalis |  | 4.0 |  | 3.9 |  | 4.0 |  | 0.5 |  | -0.02 | ***0.013****** |
|  | Pars Triangularis |  | 7.5 |  | 7.4 |  | 7.4 |  | 1.1 |  | -0.06 | ***0.004****** |
|  | Paracentral |  | 7.7 |  | 7.2 |  | 6.8 |  | 1.1 |  | -0.08 | ***0.016*** |
| Temporal Lobe | |  |  |  |  |  |  |  |  |  |  |  |
|  | Hippocampus |  | 7.6 |  | 7.5 |  | 7.2 |  | 0.8 |  | -0.08 | ***0.001****** |
|  | Parahippocampus |  | 4.0 |  | 4.0 |  | 4.0 |  | 0.5 |  | -0.01 | *0.286* |
|  | Amygdala |  | 3.3 |  | 3.1 |  | 3.0 |  | 0.3 |  | -0.03 | ***0.001****** |
|  | Fusiform gyrus |  | 15.6 |  | 14.5 |  | 14.8 |  | 2.5 |  | -0.11 | ***0.013****** |
|  | Superior temporal |  | 30.6 |  | 29.4 |  | 29.6 |  | 3.5 |  | -0.26 | ***0.001****** |
|  | Middle temporal |  | 26.3 |  | 24.8 |  | 25.4 |  | 4.1 |  | -0.30 | ***0.001****** |
|  | Inferior temporal |  | 24.3 |  | 23.3 |  | 23.8 |  | 2.8 |  | -0.11 | ***0.007****** |
| Parietal Lobe | |  |  |  |  |  |  |  |  |  |  |  |
|  | Postcentral |  | 19.5 |  | 19.1 |  | 18.1 |  | 2.1 |  | -0.08 | *0.159* |
|  | Superior parietal |  | 20.4 |  | 19.2 |  | 17.8 |  | 3.2 |  | -0.36 | ***0.001****** |
|  | Inferior parietal |  | 25.6 |  | 22.8 |  | 21.8 |  | 4.3 |  | -0.57 | ***0.001****** |
|  | Supramarginal |  | 18.1 |  | 17.6 |  | 17.7 |  | 2.3 |  | -0.09 | *0.069* |
|  | Precuneus |  | 17.7 |  | 16.6 |  | 16.7 |  | 2.4 |  | -0.17 | ***0.002****** |
| Occipital Lobe | |  |  |  |  |  |  |  |  |  |  |  |
|  | Pericalcarine |  | 3.8 |  | 3.5 |  | 3.5 |  | 1.1 |  | -0.09 | ***0.001****** |
|  | Cuneus |  | 7.3 |  | 7.0 |  | 6.8 |  | 1.2 |  | -0.11 | ***0.001****** |
|  | Lingual |  | 12.7 |  | 12.0 |  | 11.9 |  | 2.0 |  | -0.15 | ***0.001****** |
|  | Lateral occipital |  | 25.3 |  | 22.1 |  | 20.6 |  | 3.5 |  | -0.11 | *0.331* |
| Insula and Cingulate Gyri | |  |  |  |  |  |  |  |  |  |  |  |
|  | Insula |  | 5.9 |  | 5.8 |  | 6.2 |  | 1.2 |  | 0.01 | *0.739* |
|  | Anterior Cingulate |  | 11.2 |  | 10.8 |  | 11.6 |  | 1.7 |  | -0.02 | *0.614* |
|  | Isthmus Cingulate |  | 4.5 |  | 4.6 |  | 4.7 |  | 0.7 |  | 0.03 | ***0.013****** |
|  | Posterior cingulate |  | 6.2 |  | 5.9 |  | 6.1 |  | 1.0 |  | -0.04 | *0.058* |
| Central Structures | |  |  |  |  |  |  |  |  |  |  |  |
|  | Caudate |  | 6.7 |  | 6.5 |  | 6.4 |  | 0.5 |  | -0.03 | ***0.005****** |
|  | Putamen |  | 8.8 |  | 8.7 |  | 8.6 |  | 0.8 |  | -0.04 | ***0.002****** |
|  | Thalamus |  | 14.2 |  | 14.3 |  | 14.0 |  | 0.9 |  | 0.01 | *0.369* |

P-value significant indicated in bold (*p* ≤ 0.05); * statistically significant after FDR correction (*p*< 0.05).

Supplementary Table 4: Regional brain cortical thickness (mm) over the four year follow-up.

| **Regions of interest** | |  | **T0** |  | **T2** |  | **T4** |  | **SEM** |  | ***Fixed Effects Estimation*** | ***p-value*** |
| --- | --- | --- | --- | --- | --- | --- | --- | --- | --- | --- | --- | --- |
|  |  |  | **Mean** |  | **Mean** |  | **Mean** |  |  |  |  |  |
| Frontal Lobe | |  |  |  |  |  |  |  |  |  |  |  |
|  | Precentral |  | 2.23 |  | 2.07 |  | 1.97 |  | 0.23 |  | -0.01 | *0.116* |
|  | Superior frontal |  | 2.55 |  | 2.39 |  | 2.33 |  | 0.21 |  | -0.01 | *0.152* |
|  | Caudal Middle frontal |  | 2.48 |  | 2.33 |  | 2.25 |  | 0.22 |  | -0.01 | *0.072* |
|  | Rostral Middle frontal |  | 2.37 |  | 2.22 |  | 2.18 |  | 0.17 |  | -0.01 | *0.069* |
|  | Pars Opercularis |  | 2.56 |  | 2.41 |  | 2.39 |  | 0.21 |  | -0.02 | ***0.048*** |
|  | Pars Orbitalis |  | 2.68 |  | 2.55 |  | 2.54 |  | 0.18 |  | -0.01 | *0.064* |
|  | Pars Triangularis |  | 2.45 |  | 2.31 |  | 2.24 |  | 0.18 |  | -0.02 | ***0.010****** |
|  | Paracentral |  | 2.12 |  | 1.97 |  | 1.81 |  | 0.25 |  | -0.01 | *0.177* |
| Temporal Lobe | |  |  |  |  |  |  |  |  |  |  |  |
|  | Parahippocampus |  | 2.82 |  | 2.71 |  | 2.71 |  | 0.26 |  | -0.02 | ***0.008****** |
|  | Fusiform gyrus |  | 2.66 |  | 2.53 |  | 2.50 |  | 0.22 |  | -0.02 | ***0.018*** |
|  | Superior temporal |  | 2.84 |  | 2.68 |  | 2.65 |  | 0.21 |  | -0.03 | ***0.001****** |
|  | Middle temporal |  | 2.81 |  | 2.68 |  | 2.68 |  | 0.19 |  | -0.01 | *0.104* |
|  | Inferior temporal |  | 2.83 |  | 2.83 |  | 2.87 |  | 0.17 |  | +0.01 | *0.703* |
| Parietal Lobe | |  |  |  |  |  |  |  |  |  |  |  |
|  | Postcentral |  | 1.97 |  | 1.87 |  | 1.79 |  | 0.14 |  | -0.01 | ***0.013****** |
|  | Superior parietal |  | 2.15 |  | 1.99 |  | 1.91 |  | 0.18 |  | -0.01 | *0.299* |
|  | Inferior parietal |  | 2.47 |  | 2.29 |  | 2.24 |  | 0.20 |  | -0.01 | *0.271* |
|  | Supramarginal |  | 2.51 |  | 2.38 |  | 2.37 |  | 0.17 |  | -0.01 | *0.121* |
|  | Precuneus |  | 2.34 |  | 2.19 |  | 2.16 |  | 0.19 |  | -0.02 | ***0.001****** |
| Occipital Lobe | |  |  |  |  |  |  |  |  |  |  |  |
|  | Pericalcarine |  | 1.52 |  | 1.40 |  | 1.34 |  | 0.13 |  | 0.00 | *0.233* |
|  | Cuneus |  | 1.85 |  | 1.68 |  | 1.60 |  | 0.19 |  | -0.01 | *0.122* |
|  | Lingual |  | 2.03 |  | 1.88 |  | 1.82 |  | 0.17 |  | -0.01 | *0.247* |
|  | Lateral occipital |  | 2.04 |  | 2.11 |  | 2.02 |  | 0.15 |  | +0.01 | *0.621* |
| Insula and Cingulate Gyri | |  |  |  |  |  |  |  |  |  |  |  |
|  | Insula |  |  |  |  |  |  |  |  |  |  |  |
|  | Anterior Cingulate |  | 2.72 |  | 2.66 |  | 2.72 |  | 0.22 |  | +0.01 | *0.601* |
|  | Isthmus Cingulate |  | 2.33 |  | 2.26 |  | 2.26 |  | 0.19 |  | -0.01 | *0.257* |
|  | Posterior cingulate |  | 2.47 |  | 2.37 |  | 2.36 |  | 0.18 |  | -0.01 | *0.203* |

P-value significant indicated in bold (*p* ≤ 0.05); * statistically significant after FDR correction (*p*< 0.05).

Supplementary Table 5: Relationships between cognition and brain function (K_glc_, K_acac_) and volume in caudate region.

| **Cognition (raw scores)** | **K_glc_ in caudate** | | |  | **K_acac_ in caudate** | | |  | **Volume of caudate** | | |
| --- | --- | --- | --- | --- | --- | --- | --- | --- | --- | --- | --- |
|  | r | | *p* |  | r | | *p* |  | r | | *p* |
| MMSE | - | 0.04 | *0.854* |  | + | 0.20 | *0.357* |  | + | 0.11 | *0.617* |
| MOCA | + | 0.17 | *0.437* |  | + | 0.40 | *0.056* |  | - | 0.11 | *0.597* |
| WMS-III Letter-Number Sequencing | + | 0.33 | *0.112* |  | + | 0.25 | *0.247* |  | + | 0.10 | *0.642* |
| Rey Complex Figure Test - Immediate Recall | + | 0.07 | *0.756* |  | + | 0.22 | *0.309* |  | + | 0.08 | *0.723* |
| Rey Complex Figure Test - Delay Recall | + | 0.03 | *0.876* |  | + | 0.14 | *0.503* |  | + | 0.39 | *0.063* |
| D-KEFS Stroop Inhibition/Switching | - | 0.39 | *0.058* |  | + | 0.41 | ***0.044*** |  | - | 0.08 | *0.711* |
| D-KEFS Verbal Fluency Letter | - | 0.01 | *0.963* |  | + | 0.54 | ***0.007*** |  | - | 0.08 | *0.726* |
| D-KEFS Verbal Fluency Category | - | 0.09 | *0.690* |  | + | 0.33 | *0.115* |  | + | 0.23 | *0.276* |
| D-KEFS Verbal Fluency Category Switching | + | 0.13 | *0.554* |  | + | 0.31 | *0.142* |  | - | 0.05 | *0.821* |
| WAIS-IV Symbol Search | + | 0.33 | *0.113* |  | + | 0.04 | *0.839* |  | - | 0.05 | *0.832* |
| WAIS-IV Code | + | 0.31 | *0.146* |  | + | 0.06 | *0.776* |  | - | 0.14 | *0.517* |
| Boston Naming Test | + | 0.12 | *0.592* |  | + | 0.35 | *0.091* |  | + | 0.07 | *0.739* |
| WMS-III Verbal Paired Associated I Recall | - | 0.10 | *0.646* |  | + | 0.45 | ***0.029*** |  | + | 0.19 | *0.366* |
| WMS-III Verbal Paired Associated II Delay Recall | - | 0.11 | *0.617* |  | + | 0.28 | *0.187* |  | + | 0.14 | *0.512* |
| WMS-III Spatial Span Backward | + | 0.05 | *0.809* |  | + | 0.05 | *0.823* |  | + | 0.09 | *0.687* |
| WMS-III Logical Memory I Recall | + | 0.04 | *0.841* |  | + | 0.04 | *0.843* |  | + | 0.20 | *0.346* |
| WMS-III Logical Memory II Delay Recall | - | 0.17 | *0.415* |  | + | 0.15 | *0.473* |  | + | 0.31 | *0.137* |
| WAIS-IV Digit Span Backward | + | 0.41 | ***0.044*** |  | + | 0.22 | *0.301* |  | + | 0.53 | ***0.007*** |
| WAIS-IV Digit Span Sequencing | + | 0.32 | *0.132* |  | + | 0.26 | *0.229* |  | + | 0.13 | *0.553* |
| D-KEFS Trail Making Visual Scanning | + | 0.43 | ***0.034*** |  | + | 0.01 | *0.974* |  | + | 0.11 | *0.624* |
| D-KEFS Trail Number Sequence | - | 0.35 | *0.095* |  | - | 0.09 | *0.675* |  | - | 0.07 | *0.747* |
| D-KEFS Trail Letter Sequence | - | 0.20 | *0.345* |  | - | 0.26 | *0.222* |  | + | 0.00 | *0.985* |
| D-KEFS Trail Number-Letter Sequence | - | 0.25 | *0.238* |  | - | 0.08 | *0.727* |  | + | 0.01 | *0.981* |
| Executive function (Composite z-score) | + | 0.26 | *0.214* |  | + | 0.34 | *0.102* |  | + | 0.02 | *0.912* |
| Working memory (Composite z-score) | + | 0.18 | *0.392* |  | + | 0.26 | *0.221* |  | + | 0.34 | *0.102* |
| Episodic memory Immediate + delay recall (Composite z-score) | - | 0.10 | *0.644* |  | + | 0.35 | *0.095* |  | + | 0.29 | *0.165* |
| Language (Composite z-score) | + | 0.04 | *0.861* |  | + | 0.38 | *0.067* |  | + | 0.26 | *0.221* |
| Attention and processing speed (Composite z-score) | + | 0.36 | *0.081* |  | + | 0.17 | *0.439* |  | + | 0.06 | *0.794* |

P-value significant indicated in bold (*p* ≤ 0.05)
